# Complement Is Activated During Normothermic Machine Perfusion of Porcine and Human Discarded Kidneys

**DOI:** 10.3389/fimmu.2022.831371

**Published:** 2022-07-13

**Authors:** Neeltina M. Jager, Leonie H. Venema, Asel S. Arykbaeva, Anita H. Meter-Arkema, Petra J. Ottens, Cees van Kooten, Tom E. Mollnes, Ian P. J. Alwayn, Henri G. D. Leuvenink, Soeren E. Pischke, PROPER study consortium

**Affiliations:** ^1^ Department of Surgery, University Medical Center Groningen, Groningen, Netherlands; ^2^ LUMC Transplant Center, Department of Surgery, Leiden University Medical Center, Leiden, Netherlands; ^3^ Department of Nephrology, University Medical Center Groningen, Groningen, Netherlands; ^4^ LUMC Transplant Center, Department of Nephrology, Leiden University Medical Center, Leiden, Netherlands; ^5^ Department of Immunology, University of Oslo and Oslo University Hospital Rikshospitalet, Oslo, Norway; ^6^ Research Laboratory, Nordland Hospital, Bodø, Norway; ^7^ K.G. Jebsen Thrombosis Research and Expertise Center, University of Tromsø, Tromsø, Norway; ^8^ Center of Molecular Inflammation Research, Norwegian University of Science and Technology, Trondheim, Norway; ^9^ Department of Anaesthesiology and Intensive Care, Oslo University Hospital, Oslo, Norway

**Keywords:** kidney transplantation, normothermic machine perfusion, complement system, ischemia-reperfusion injury, immunology

## Abstract

**Background:**

The gap between demand and supply of kidneys for transplantation necessitates the use of kidneys from extended criteria donors. Transplantation of these donor kidneys is associated with inferior results, reflected by an increased risk of delayed graft function. Inferior results might be explained by the higher immunogenicity of extended criteria donor kidneys. Normothermic machine perfusion (NMP) could be used as a platform to assess the quality and function of donor kidneys. In addition, it could be useful to evaluate and possibly alter the immunological response of donor kidneys. In this study, we first evaluated whether complement was activated during NMP of porcine and human discarded kidneys. Second, we examined the relationship between complement activation and pro-inflammatory cytokines during NMP. Third, we assessed the effect of complement activation on renal function and injury during NMP of porcine kidneys. Lastly, we examined local complement C3d deposition in human renal biopsies after NMP.

**Methods:**

NMP with a blood-based perfusion was performed with both porcine and discarded human kidneys for 4 and 6 h, respectively. Perfusate samples were taken every hour to assess complement activation, pro-inflammatory cytokines and renal function. Biopsies were taken to assess histological injury and complement deposition.

**Results:**

Complement activation products C3a, C3d, and soluble C5b-9 (sC5b-9) were found in perfusate samples taken during NMP of both porcine and human kidneys. In addition, complement perfusate levels positively correlated with the cytokine perfusate levels of IL-6, IL-8, and TNF during NMP of porcine kidneys. Porcine kidneys with high sC5b-9 perfusate levels had significantly lower creatinine clearance after 4 h of NMP. In line with these findings, high complement perfusate levels were seen during NMP of human discarded kidneys. In addition, kidneys retrieved from brain-dead donors had significantly higher complement perfusate levels during NMP than kidneys retrieved from donors after circulatory death.

**Conclusion:**

Normothermic kidney machine perfusion induces complement activation in porcine and human kidneys, which is associated with the release of pro-inflammatory cytokines and in porcine kidneys with lower creatinine clearance. Complement inhibition during NMP might be a promising strategy to reduce renal graft injury and improve graft function prior to transplantation.

## Introduction

**Figure 1:**
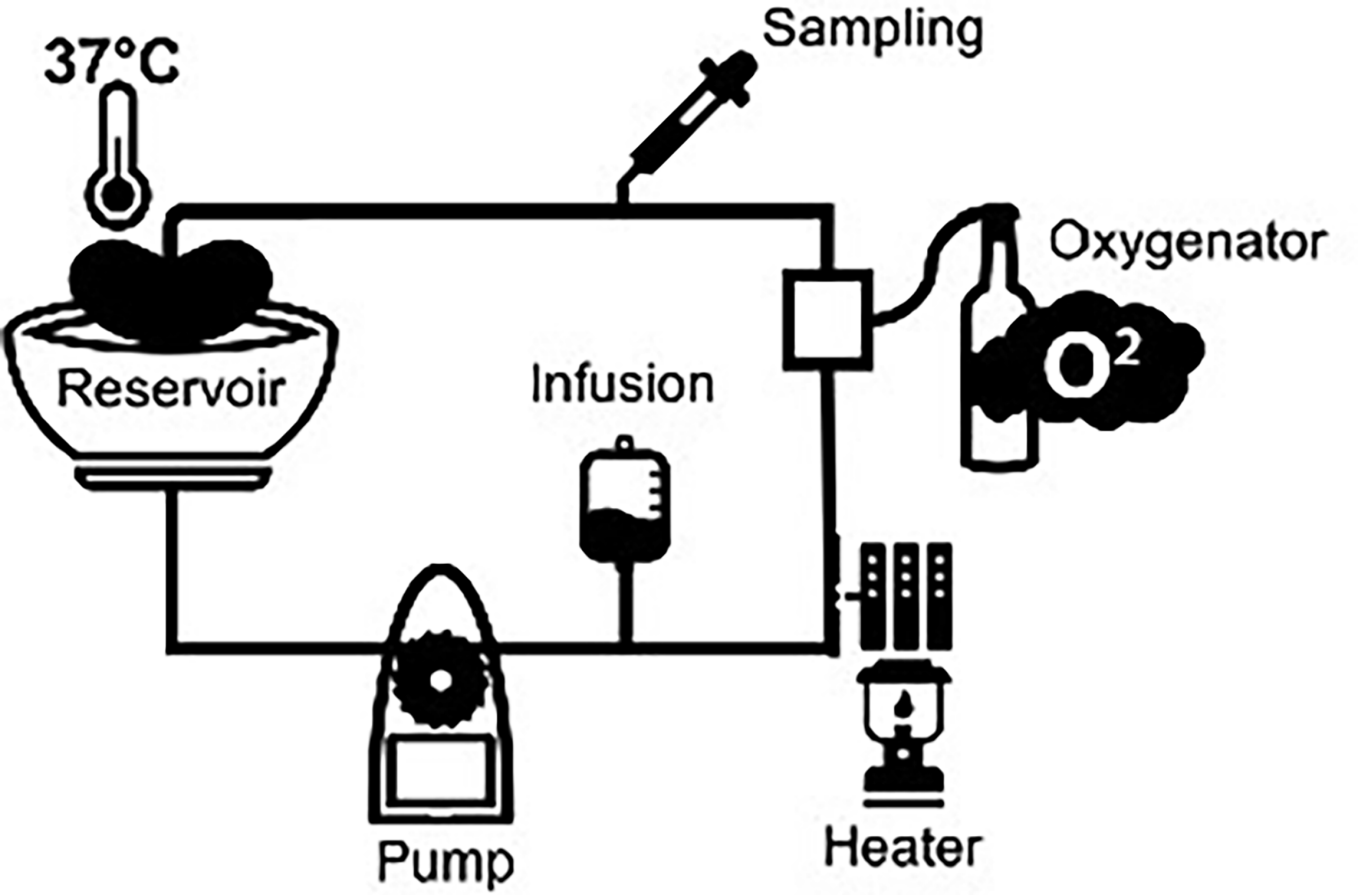
Normothermic machine perfusion setup.

Normothermic machine perfusion (NMP) is a preservation technique that recently has been introduced to assess organ quality prior to transplantation. The increasing gap between demand and supply resulted in the necessity to transplant kidneys with a lower graft quality and has resulted in an increased risk of delayed graft function and inferior renal function compared to standard criteria donor kidneys (1, 2). The exact mechanism is unknown, but it is hypothesized that the higher immunogenicity of older donors and increased comorbidities lead to inferior results after transplantation. The increased use of extended criteria donor kidneys led to the idea of tailoring the preservation method to the renal graft, which resulted in the use of NMP. During NMP, the renal graft is perfused with an oxygenated perfusion solution at 37°C (**Figure 1**). Maintaining a kidney at a normothermic temperature has many advantages. It not only provides the possibility of evaluating kidney quality but also reconditions prior to transplantation. Furthermore, it would be possible to characterize immunological responses of donor kidneys. Limited studies have shown the potential of machine perfusion to reduce the immune response in the donor kidney. Pig studies showed reduced graft immunogenicity by initiating an inflammatory cytokine storm leading to leukocyte mobilization and removal prior to renal transplantation. NMP of porcine kidneys resulted in the modulation of pro-inflammatory gene expression levels and a decrease in the number of leukocytes in the kidney prior to transplantation (3, 4). However, less is known about the pathophysiology of these immunological responses and how to reduce the immunogenicity of kidneys during NMP.

Kidneys are equipped with a sophisticated localized immune system. Previous studies have shown that severe injury, such as brain death or ischemia–reperfusion injury, induces a robust local immune response. As part of the local immune response, the complement system is activated (3, 5–7). To avoid inappropriate complement activation under normal conditions, complement is strictly regulated by regulatory proteins. Inappropriate activation of the complement system can have deleterious effects on kidneys and needs to be prevented when possible (8, 9). It is known that the complement system can be activated through contact with foreign surfaces, which is the case during extracorporeal membrane oxygenation (ECMO), cardiopulmonary bypass (CPB), and hemodialysis (HD) (10, 11). An NMP system consists of the same components as an ECMO, CPB, and HD system. Therefore, it is likely that NMP also results in complement activation. In addition, complement activation also plays a prominent role in renal graft injury, which is started in the donor and continued in the recipient (12, 13). Given the importance of complement in renal graft injury, modulating the complement system is a potential promising strategy to improve renal transplant outcome (13, 14). This study aimed to examine whether complement gets activated during NMP of porcine and human discarded kidneys. The effect of complement activation on the pro-inflammatory response and renal function during NMP of porcine kidneys was investigated.

## Materials and Methods

### Normothermic Machine Perfusion of Porcine Kidneys

In the preclinical phase, porcine kidneys (from female, Dutch landrace pigs) and autologous heparinized whole blood were used, as described previously (15). In short, kidneys (*n* = 20) were exposed to a warm ischemia time of 30 min to induce ischemic injury. Subsequently, kidneys were cold flushed with 180 ml of cold (4°C) saline (Baxter BV, Utrecht, The Netherlands) and stored for 24 h by either static cold storage (SCS) or with hypothermic machine perfusion (HMP) with a mean arterial pressure of 25 mmHg with no active oxygenation or with 21% or 100% oxygen (15). Kidneys of the HMP groups were cannulated for connection to the HMP device (Kidney Assist Transport, Organ Assist, Groningen, The Netherlands) and perfused with 500 ml of University of Wisconsin machine perfusion solution (UW-MP solution, Bridge to Life Ltd., London, United Kingdom). SCS preserved kidneys were submerged in 500 ml of University of Wisconsin cold storage solution (UW-CS, Bridge to Life Ltd.) and stored on ice. Next, kidneys were flushed with 50 ml of cold saline solution to remove the remaining UW solution and connected to the NMP device (**Figure 1**). Thereafter, all kidneys were pressure-controlled perfused with a mean arterial pressure of 75 mmHg at a normothermic temperature (37°C) for 4 h (Kidney Assist Transport, Organ Assist). The perfusion solution was leukocyte-depleted blood diluted with Ringer’s lactate solution and several additives (**Table 1**). Renal artery and ureter were cannulated with a 12F and 8F cannula respectively. The perfusion solution was oxygenated *via* an oxygenator (Medos Medizin AG, Stolberg, Germany) with a mixture of 95% O_2_/5% CO_2_ at a flow rate of 0.5 L/min. During NMP, there was a continuous supply of nutrients, which was administered at a rate of 20 ml/h (**Table 1**). In case the glucose levels dropped below 5 mmol/L, glucose 5% was administered. To differentiate between the activation of complement by the kidney versus by the machine setup, i.e., tubing and oxygenator, we included a small (*n* = 2) kidney-free NMP control group where NMP was performed with heparinized pig blood for 4 h without a kidney.

**Table 1:** Composition of the perfusion solution used for normothermic machine perfusion.

Perfusion solution—Porcine kidneys	Perfusion solution—Human discarded kidneys
500 ml of Leukocyte-depleted blood	2 packed washed red blood cells
1,000 mg/200 mg of Amoxicillin/Clavulanate	1000 mg of Cefazoline
6 mg Mannitol	20 ml of 15% Mannitol
10 ml of 8.4% Sodium bicarbonate	Approximately 15–20 ml 8.4% Sodium bicarbonate, to correct pH before perfusion
100 µl of 20 mg/ml Sodium Nitroprusside	20 ml of 10% Calcium Gluconate
300 ml of Ringer’s lactate	500 ml of NaCl
90 mg of Creatinine	100 ml of 20% Albumin
6 mg of Dexamethasone	
Infusion solution—Porcine kidneys	Infusion solution—Human discarded kidneys
80 ml of 10% Aminoplasmal	50 ml of 10% Aminoplasmal [23.3 ml/h]
17 IU Novorapid	Cernevit multivitamins (0.5 ml added to aminoplasmal)
	0.5 mg of Flolan [5.8 ml/h]
	Glucose 5% [8 ml/h]

### Normothermic Machine Perfusion of Human Discarded Kidneys

Human discarded kidneys were included within the Prolonged *ex situ* NMP for kidney regeneration (PROPER) trial registered in the Dutch Trial Register as NL8446. The PROPER trial is a Dutch initiative from three University transplant centers—The University Medical Center Groningen (UMCG), Leiden University Medical Center (LUMC), and Rotterdam Erasmus Medical Center (Erasmus MC)—to introduce and clinically evaluate prolonged NMP. As part of the PROPER trial, the current study investigated the role of complement activation during NMP in human discarded kidneys. The ten analyzed kidneys were included between January 1, 2019 and August 1, 2019. The included kidneys were retrieved from donation after brain death (DBD) and donation after circulatory death (DCD) donors, but discarded postretrieval. Reasons for discarding are described in **Table 3**. Kidneys were cold flushed with University of Wisconsin solution (UW-CS, Bridge to Life Ltd.) and preserved *via* SCS or with HMP. As a perfusion solution, matched banked red blood cells (RBCs) were used, which were washed prior to NMP with 2 L of 0.9% NaCl by using a CellSaver (Fresenius C.A.T.S. plus, Fresenius Kabi GmbH, Bad Homburg, Germany) and afterwards diluted with 0.9% NaCl (**Table 1**). Upon arrival, the renal artery and ureter were cannulated with a 12F and 8F cannula, respectively. After preparation, the kidney was weighed and flushed with 200 ml of Ringer’s lactate solution and connected to the NMP device (Kidney Assist Transport, Organ Assist). Kidneys were pressure-controlled perfused with a mean arterial pressure of 75 mmHg for 6 h. The perfusion solution was oxygenated *via* an oxygenator (Medos Medizin AG) with a mixture of 95% O_2_/5% CO_2_ at a flow rate of 0.5 L/min. During NMP, there was a continuous supply of nutrients. Specifics on infusion and the perfusion solution can be found in **Table 1**.

### Perfusate Samples

Perfusate samples during NMP were taken at baseline, 30 min, 1 h, 2 h, 3 h, and 4 h. Samples at time points 5 h and 6 h were only taken during NMP of human discarded kidneys. Samples were collected in EDTA tubes (Biosciences, Plymouth, UK) and stored on ice and centrifuged at 4°C, 2,500 × *g* for 20 min. Plasma was stored at −80°C.

### Renal Function

Creatinine concentration was measured in perfusate and urine, using routine procedures at the clinical chemistry lab of the University Medical Center Groningen. Creatinine clearance (L/min/100 g) was calculated with the following formula ((urine creatinine concentration (mmol/L) * urine flow (ml/min)/perfusate creatine concentration (mmol/L))/kidney weight (g))) *100.

### Renal Morphology After NMP of Porcine Kidneys

Renal biopsies were taken after 4 h of NMP. Paraffin sections (4 μm) were stained with hematoxylin-eosin (H&E). Histological injury was scored on the basis of two criteria: proximal tubular cell necrosis and proximal tubular cell edema. Histological injury was scored blinded by two independent examiners under the supervision of a pathologist. Proximal tubular cell necrosis and proximal tubular cell edema were scored as described previously (15): (1) no necrosis/edema; (2) minor signs of necrosis/edema; (3) moderate signs of necrosis/edema; (4) severe necrosis/edema; and (5) complete necrosis/edema. The different biopsies were randomly assigned to the examiners, and in case of discrepancy, the supervising pathologist was consulted.

### Renal C3d Deposition After NMP of Human Discarded Kidneys

Renal biopsies were taken after 6 h of NMP. Frozen sections (4 μm) were fixed with acetone and endogenous peroxidase was blocked by incubating all sections in 30% H_2_O_2_. Next, the sections were incubated with primary antibody against C3d (Clone A0063, Dako, CA, USA). Subsequently, sections were incubated with a goat-anti-rabbit-FITC antibody. Finally, sections were incubated with 4’ 6-diamidino-2-phenylindole (DAPI) to stain the nuclei and mounted with Citifluor. Tissue images were acquired using Leica confocal microscope (Leica, Wetzlar, Germany). Sections were quantified by two independent examiners. An immunofluorescence density (ID) score was calculated based on (1) the intensity of the staining in the renal cortex (scored from 1 to 5) and (2) the percentage area of the renal cortex with C3d deposition (scored from 0 to 100).

### Complement Assays for Perfusate Samples After NMP of Porcine Kidneys

To measure complement activation products in the perfusate samples, complement activation products at the level of C3 were measured. In the perfusate samples retrieved from NMP with porcine kidneys, C3a was measured using a highly specific porcine ELISA described earlier, using antibodies reacting with the C3a fragment (16). The terminal pathway activation was measured by soluble C5b-9 (sC5b-9) using the monoclonal antibody aE11 as capture antibody (17). It reacts efficiently with a neoepitope exposed in both human- and pig-activated C9, and the assay was performed as described previously. The level was related to the International Complement Standard #2, defined to contain 1,000 complement arbitrary units (AU) per ml.

### Complement Assays for Perfusate Samples After NMP of Human Discarded Kidneys

C3 and C3d were measured in perfusate samples after NMP of human discarded kidneys. C3d was measured as described previously (18). Briefly, samples were polyethylene glycol (PEG) precipitated. PEG precipitation is necessary since free C3d shares epitopes with intact C3. Based on the measured values, a C3d/C3 ratio was calculated, which is a measure for complement activation at the level of complement C3. All samples were 1:1 diluted with 22% PEG in 0.1 M borate/EDTA buffer (pH 8.32) and incubated on ice for 3 h. Afterwards, they were centrifuged and supernatants were used for C3d quantification. Rabbit anti-human C3d was used as coating antibody (Dako). A rabbit anti-human C3d-DIG was used as capture antibody and a rabbit anti-DIG-HRP (Roche) was used as detection antibody. All samples were measured in duplicate. Values are expressed as ng/ml. A standard curve was made in a 6-fold dilution and reference samples was made from stock solution.

### Quantification of Porcine and Human Cytokines

To detect and quantify pro-inflammatory cytokines, tumor necrosis factor (TNF), interleukin-6, and interleukin-8 in the perfusate samples of porcine kidneys, we used commercial porcine immunoassay kits according to the manufacturer’s instructions (R&D Systems, Minneapolis, MN, USA). For the detection and quantification of interleukin-6 and interleukin-8 in perfusate samples of human kidneys, we used commercial human immunoassay kits according to the manufacturer’s instructions (R&D Systems, Minneapolis, MN, USA).

### Statistical Analysis

Statistical analysis was performed using GraphPad Prism Software v8.3.1. Differences between time points were tested using Kruskal–Wallis test followed by a Mann–Whitney *U post-hoc* test. Differences between two groups were tested by a Mann–Whitney *U* test. Spearman correlation coefficients were calculated to determine which cytokines were significantly associated with complement C3a and sC5b-9 levels. *P* < 0.05 was considered significant. Data are presented as median ± interquartile range (IQR).

## Results

### Complement Is Activated During Normothermic Machine Perfusion of Porcine Kidneys

**Figure 2:**
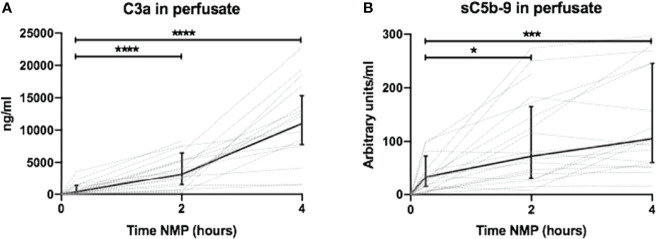
Dynamics of complement perfusate levels during 4 h of normothermic machine perfusion of porcine kidneys. Dynamics of **(A)** C3a and **(B)** sC5b-9 perfusate levels during 4 h of normothermic machine perfusion of porcine kidneys. Perfusate levels of both C3a and sC5b-9 increase during normothermic machine perfusion of porcine kidneys. Dotted lines: increase of complement perfusate levels per individual perfused kidney. Solid lines and bars: median ± interquartile range of complement perfusate levels for all kidneys (*n* = 20). *p < 0.05, ***p < 0.001, ****p < 0.0001. NMP, normothermic machine perfusion; sC5b-9, soluble C5b-9.

Complement activation was assessed by the quantification of perfusate levels of C3a and sC5b-9 during 4 h of NMP. C3a levels increased after 2 h of NMP with a steep increase to 4 h NMP and were significantly increased compared to C3a perfusate levels at 30 min of NMP (**Figure 2A**). Complement sC5b-9 perfusate levels significantly increased after 2 and 4 h of NMP (**Figure 2B**). The preservation method before NMP, hypothermic machine perfusion with or without oxygen, did not influence C3a or sC5b-9 perfusate levels during 4 h of NMP (**Supplementary Figures 1A, B**). In addition, significantly less complement was activated in kidney-free NMPs (**Supplementary Figures 2A, B**).

### High Levels of Pro-Inflammatory Cytokines Are Released Normothermic Machine Perfusion of Porcine Kidneys

**Figure 3:**
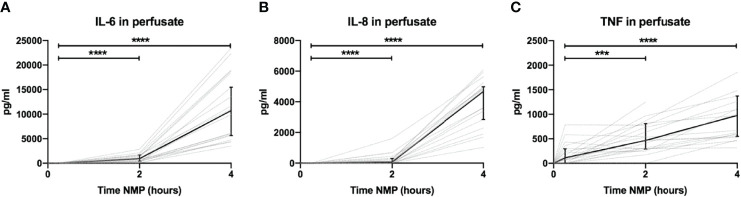
Cytokine perfusate levels during normothermic machine perfusion of porcine kidneys. Dynamics of pro-inflammatory cytokines **(A)** IL-6, **(B)** IL-8, and **(C)** TNF in perfusate during normothermic machine perfusion of porcine kidneys. Cytokine perfusate levels significantly increased during 4 h of normothermic machine perfusion. Dotted lines: increase of cytokine levels in perfusate per individual perfused kidney. Solid lines and bars: median ± interquartile range of cytokine perfusate levels for all kidneys (*n* = 20). ***p < 0.001, ****p < 0.0001. IL-6, interleukin-6; IL-8, interleukin-8; NMP, normothermic machine perfusion; TNF, tumor necrosis factor.

Perfusate levels of IL-6 and IL-8 were significantly higher after 2 and 4 h than baseline levels. Both increased exponentially between 2 and 4 h of NMP (**Figures 3A, B**, respectively). There was a significant release of TNF after 2 h of NMP, which further increased after 4 h of NMP (**Figure 3C**). TNF increased linearly throughout the NMP period. Different preservation techniques prior to NMP did not affect cytokine perfusate levels during, NMP (**Supplementary Figures 3A–C**).

### Complement and Cytokine Perfusate Levels Strongly Correlate During Normothermic Machine Perfusion of Porcine Kidneys

**Figure 4:**
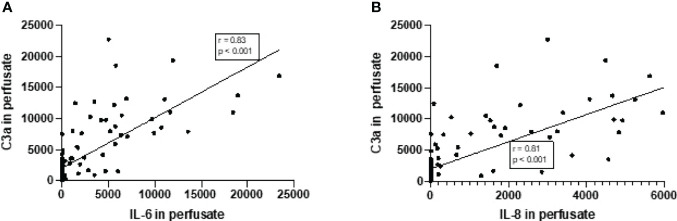
Correlation between complement C3a perfusate levels and cytokine perfusate levels in porcine kidneys after 4 h of normothermic machine perfusion. Correlation between **(A)** complement C3a and IL-6 levels in perfusate and **(B)** correlation between complement C3a and IL-8 levels in perfusate after 4 h of NMP of porcine kidneys. Spearman’s correlation coefficient (*r*) and *p*-values are indicated. IL-6, interleukin-6; IL-8, interleukin-8; NMP, normothermic machine perfusion.

Increase in complement C3a perfusate levels strongly correlated with the increase of perfusate levels of IL-6 and IL-8 (**Figures 4A, B**). In addition, there was a significant but moderate correlation between complement sC5b-9 perfusate levels and cytokine perfusate levels after 4 h of NMP (**Table 2**).

**Table 2:** Correlation between complement perfusate levels and cytokine perfusate levels after 4 h of normothermic machine perfusion of porcine kidneys.

Perfusate levels	Correlation with C3a		Correlation with sC5b-9	
	*r*	*p*-value	*r*	*p*-value
**IL-6**	0.83	<0.0001	0.52	<0.0001
**IL-8**	0.81	<0.0001	0.48	<0.0001
**TNF-alpha**	0.66	<0.0001	0.54	<0.0001

r, correlation coefficient.

### Kidneys With High sC5b-9 Perfusate Levels Have Significantly Lower Creatinine Clearance

**Figure 5:**
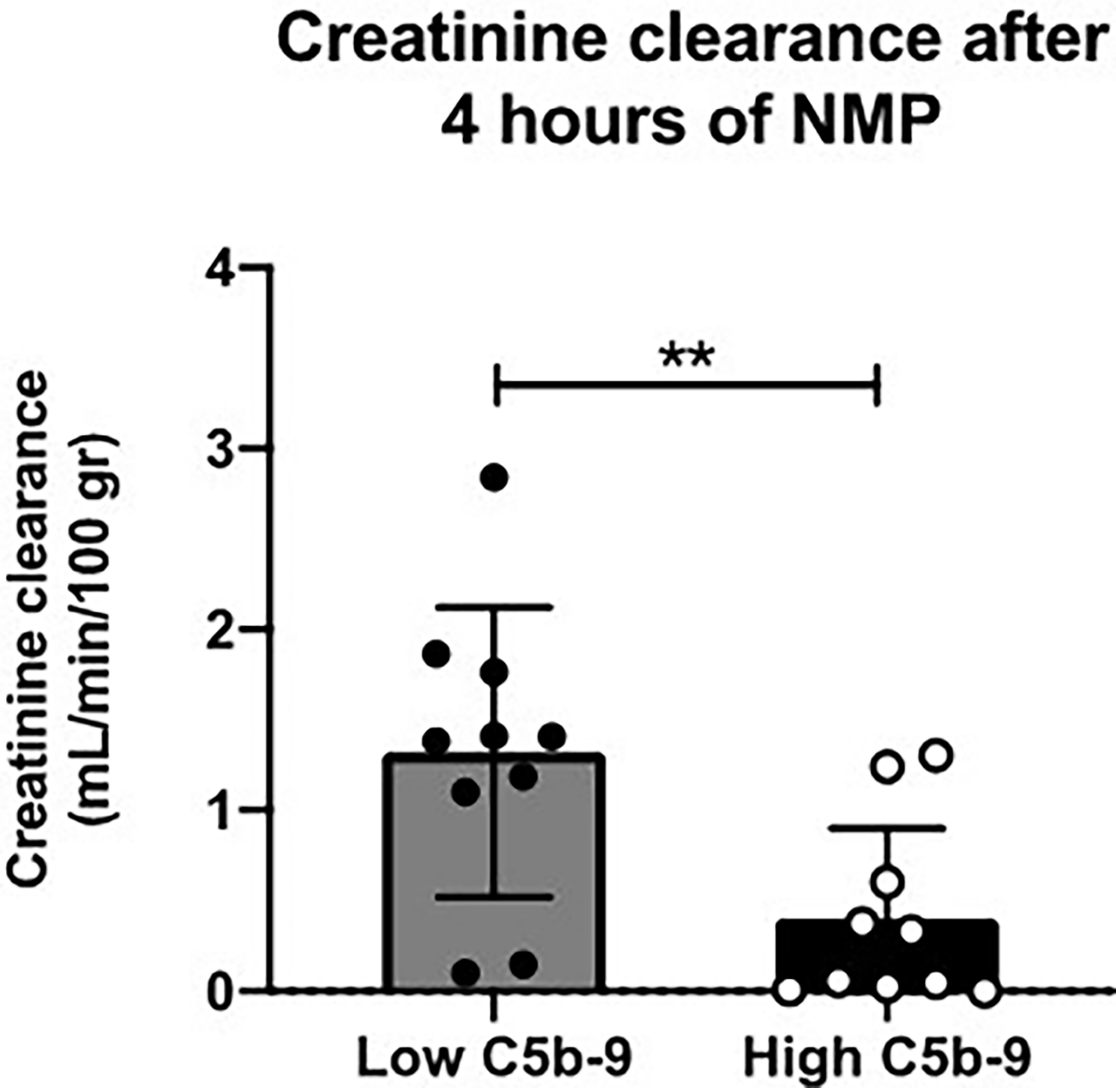
Creatinine clearance after 4 h of normothermic machine perfusion of porcine kidneys with low *versus* high sC5b-9 perfusate levels. Kidneys (*n* = 10) with low sC5b-9 perfusate levels after 4 h of normothermic machine perfusion (NMP) had significantly higher creatinine clearance than kidneys (*n* = 10) with high sC5b-9 perfusate levels after 4 h of NMP. Subgroups were based on the median sC5b-9 perfusate level after 4 h of NMP, which was 105 AU/ml. **p < 0.01. NMP, normothermic machine perfusion; sC5b-9, soluble C5b-9.

Next, subgroup analysis was performed for sC5b-9 perfusate levels after 4 h of NMP of porcine kidneys (**Figure 3B**). Two subgroups (*n* = 10/group) were formed based on the median value of sC5b-9 after 4 h of NMP (median 105 AU/ml) and the creatinine clearance for these two subgroups was calculated. Kidneys with sC5b-9 perfusate levels above 105 AU/ml after 4 h of NMP had a significant lower creatinine clearance than kidneys with sC5b-9 levels below this value (**Figure 5**).

### Porcine Kidneys With High sC5b-9 Perfusate Levels Have Significantly More Histological Injury

**Figure 6:**
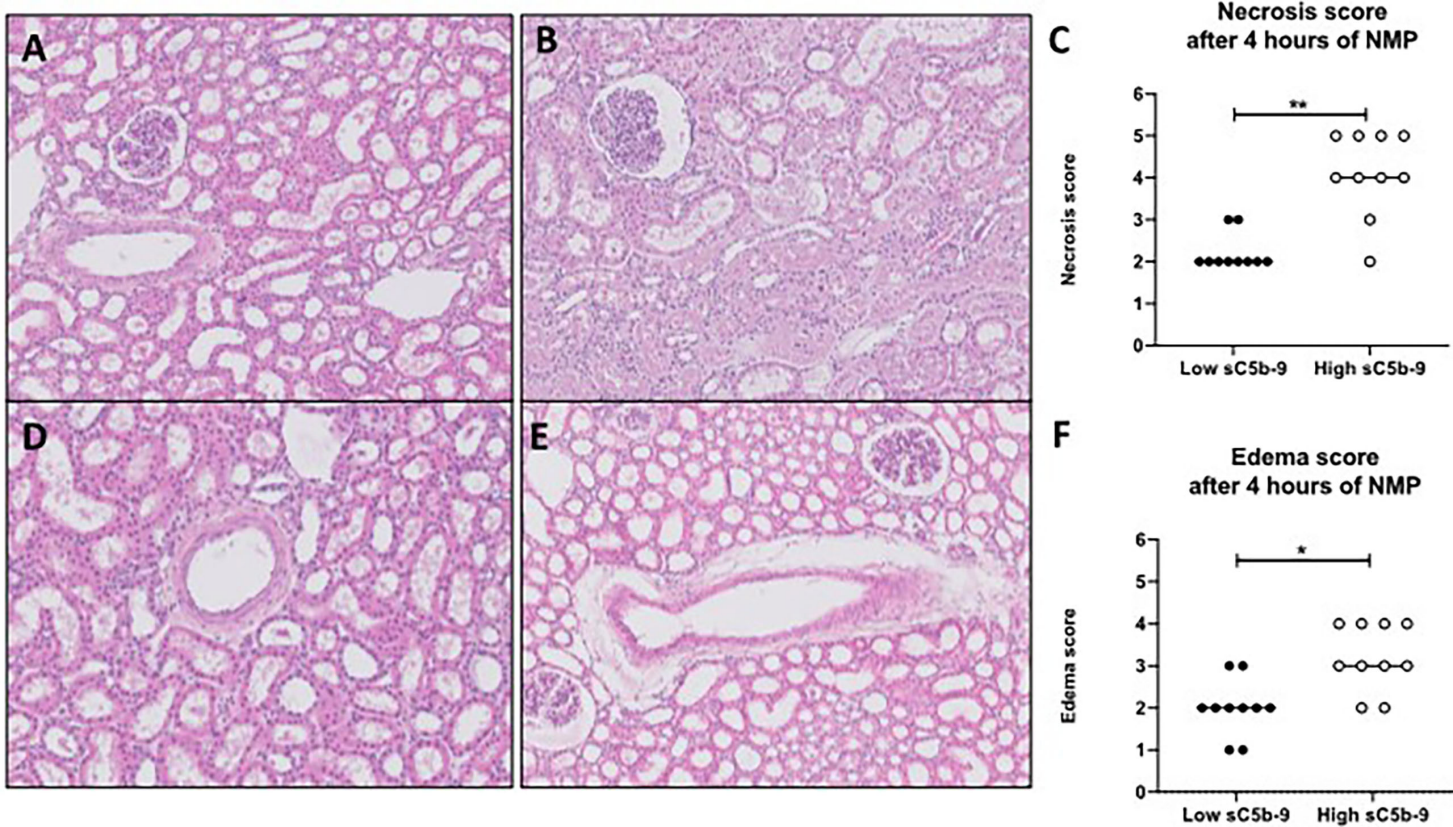
H&E-stained biopsies of porcine kidneys after 4 h of normothermic machine perfusion. **(A–C)** Proximal tubular necrosis score (scored from 1 to 5) was assessed histologically after 4 h of normothermic machine perfusion (NMP). **(A)** Representative picture of a low necrosis score (score 2) after 4 h of NMP. **(B)** Representative picture of a high necrosis score (score 5) after 4 h of NMP. **(C)** Graph of the overall necrosis score after 4 h of normothermic machine perfusion. Kidneys with low sC5b-9 perfusate levels (*n* = 10) had significantly lower necrosis scores than kidneys with high sC5b-9 perfusate levels (*n* = 10). **(D, E)** Edema score (score from 1 to 5) was assessed histologically after 4 h of NMP. **(D)** Representative picture of a low edema score (score 1). **(E)** Representative picture of a high edema score (score 4). **(F)** Graph of the overall edema score after 4 h of normothermic machine perfusion. Kidneys with low sC5b-9 perfusate levels (*n* = 10) had significantly lower edema scores than kidneys with high sC5b-9 perfusate levels (*n* = 10). Subgroups were based on the median sC5b-9 perfusate levels after 4 h of normothermic machine perfusion. *p < 0.05, **p < 0.01. Images were obtained by using Leica Confocal Software. All magnifications ×100. sC5b-9, soluble C5b-9; NMP, normothermic machine perfusion.

Histological injury was examined in porcine kidneys after 4 h of NMP. All porcine kidneys had proximal tubular cell necrosis and edema after NMP. After initial examination, the kidneys were divided in subgroups, based on low or high sC5b-9 perfusate levels (*n* = 10/group). Kidneys with high sC5b-9 perfusate levels had significantly more proximal tubular cell necrosis and edema than kidneys with low 2C5b-9 perfusate levels (**Figure 6**).

### Complement Is Activated During Normothermic Machine Perfusion of Human Discarded Kidneys

**Figure 7:**
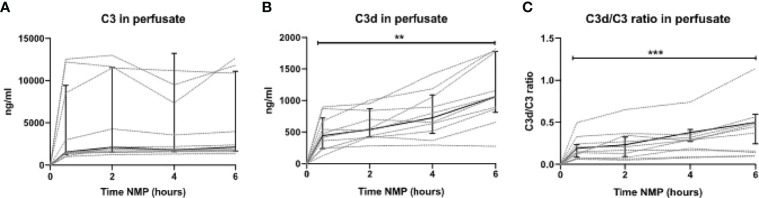
Complement perfusate levels during normothermic machine perfusion of human discarded kidneys. **(A)** Complement C3 (in ng/ml) and **(B)** C3d levels (in ng/ml) and **(C)** the calculated C3d/C3 ratio in perfusate during 6 h of normothermic machine perfusion of human discarded kidneys. Dotted lines: increase of complement per individual kidney. Solid lines and bars: median ± interquartile range for all kidneys (*n* = 10). **p < 0.01, ***p < 0.001. NMP, normothermic machine perfusion.

To translate the experimental results seen during NMP of porcine kidneys, complement activation during NMP of human discarded kidneys was investigated. These human discarded kidneys were retrieved from five DBD and five DCD donors. Baseline characteristics of these kidneys are shown in **Table 3**. To investigate whether complement was activated during NMP of human discarded kidneys, complement C3 and C3d perfusate levels were measured. Complement C3 perfusate levels did not change during 6 h of NMP (**Figure 7A**). In contrast, C3d perfusate levels significantly increased during 6 h of NMP, with the biggest increase between 4 and 6 h of NMP (**Figure 7B**). In addition, the C3d/C3 ratio significantly increased after 6 h of NMP compared to baseline levels (30 min after NMP) (**Figure 7C**).

**Table 3:** Baseline characteristics of human discarded kidneys perfused during 6 h of normothermic machine perfusion.

Characteristic	Summary
Female donor (*n*; %)	1; 10
Reason for decline (*n*; %) - Artheriosclerosis - High donor age - Dissection renal artery - High transaminase levels - Suspicious for malignancy - Medical reasons (not further specified)	2; 20 1; 10 1; 10 1; 10 1; 10 4; 40
Donor type (*n*; %) - DBD donor - DCD donor	5; 50 5; 50
Organ preservation method (*n*; %) - Static cold storage - Hypothermic machine perfusion	4; 40 6; 60
Cold ischemia time (*n*; %) - < 15 h - > 15 h	4; 40 6; 60

DBD, donation after brain death; DCD, donation after circulatory death.

### Kidneys Retrieved From Brain-Dead Donors Have Significantly Higher Complement Perfusate Levels Than Kidneys Donated After Circulatory Death

**Figure 8:**
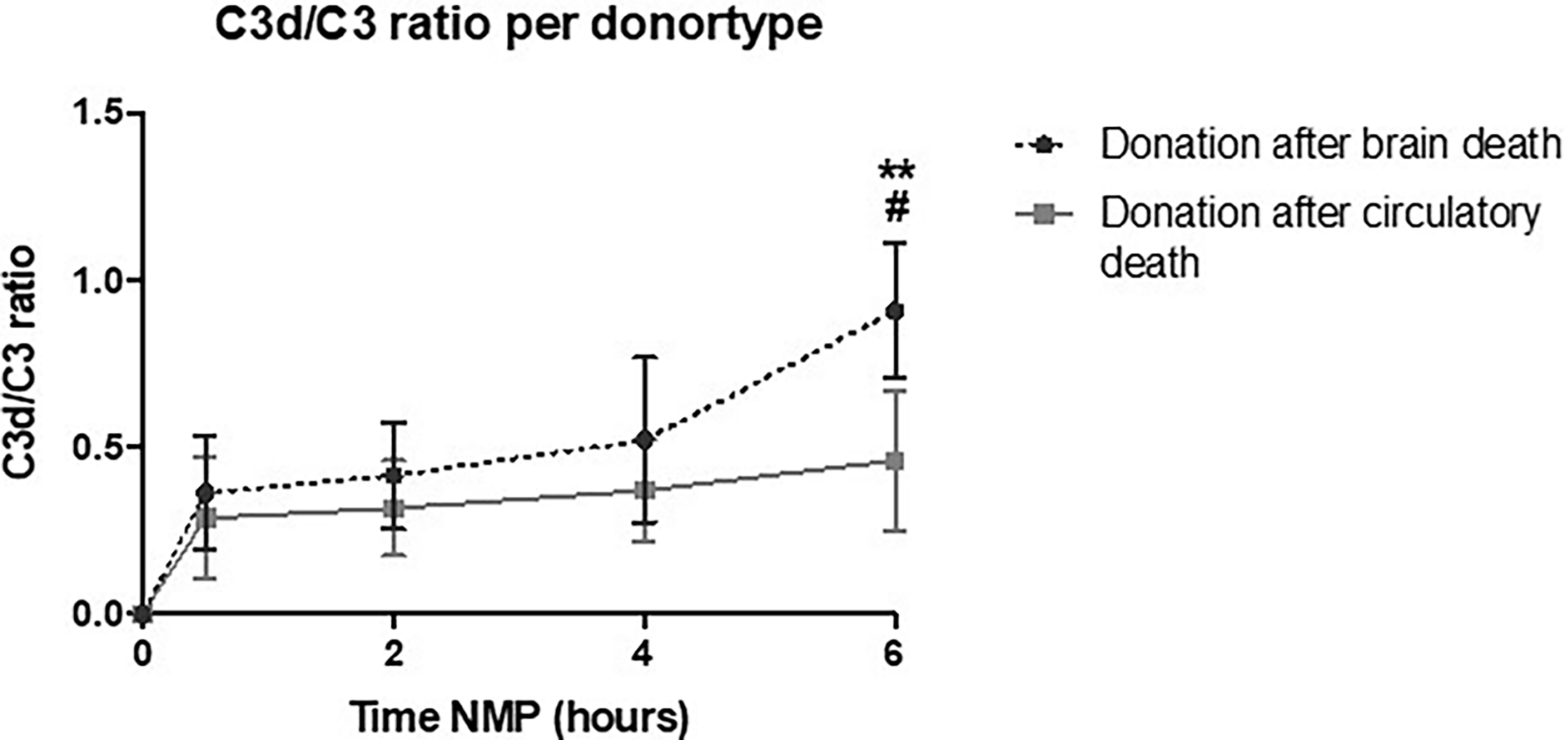
Perfusate C3d/C3 ratio of human kidneys retrieved from brain-dead donors is significantly higher after 6 h of normothermic machine perfusion. Shown are the differences in C3d/C3 ratio during normothermic machine perfusion based on donor type. Kidneys retrieved from brain-dead donors showed significantly higher C3d/C3 ratio after 6 h of normothermic machine perfusion than kidneys retrieved after circulatory death. Data shown as median ± interquartile range. The asterisk denotes a significant difference between baseline C3d/C3 ratio and C3d/C3 ratio after 6 h of NMP of kidneys from brain-dead donors. The hashtag denotes a significant difference in C3d/C3 ratio between the two groups, brain-dead donors and donation after circulatory death. #*p* < 0.05, **p < 0.01. NMP, normothermic machine perfusion.

To investigate the role of different types of donors, C3d/C3 ratio during NMP of kidneys retrieved from DBD *versus* DCD donors were compared. Kidneys retrieved from DBD donors had a significantly higher C3d/C3 ratio at 6 h of NMP than kidneys retrieved from DCD donors (**Figure 8**). During NMP of kidneys retrieved from DBD donors, there was a significant increase of the C3d/C3 ratio over time. In contrast, the C3d/C3 ratio during NMP of kidneys from DCD donors did not significantly change over time. No differences in the C3d/C3 ratio were seen based on different preservation methods (SCS *versus* HMP) or based on the cold ischemia time (shorter *versus* longer than 15 h) (data are not shown).

### Kidneys Retrieved From Brain-Dead Donors Have Significantly More Renal C3d Deposition

**Figure 9:**
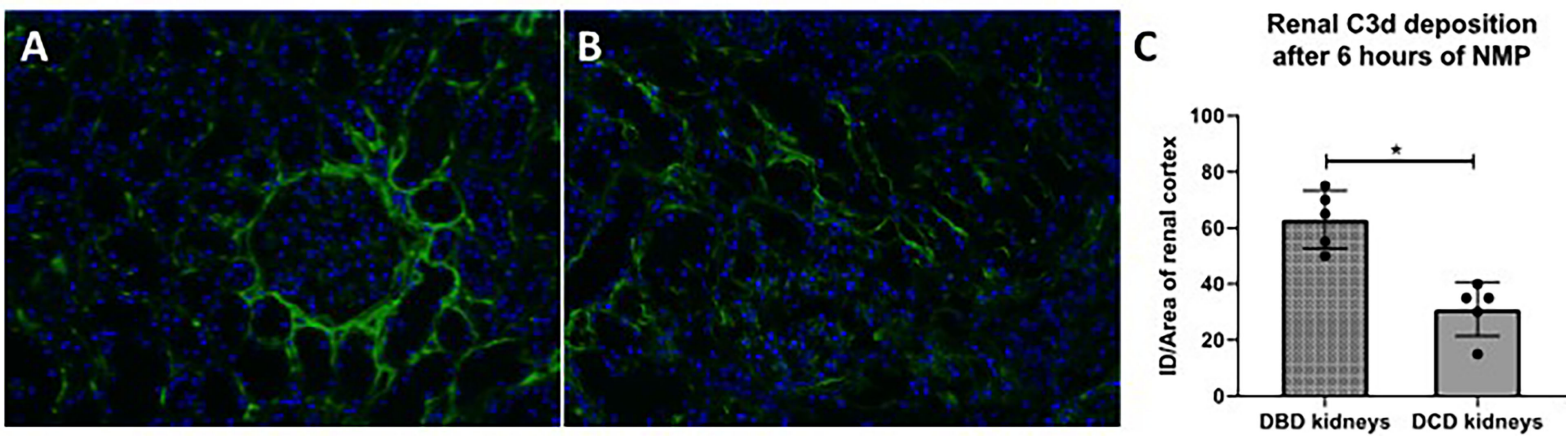
C3d deposition in human discarded kidneys after 6 h of normothermic machine perfusion. Confocal microscopy of human discarded kidneys procured from **(A)** brain-dead donor and **(B)** a circulatory death donor after 6 h of normothermic machine perfusion. **(C)** Difference in C3d deposition after 6 h of normothermic machine perfusion in kidneys derived from brain-dead versus circulatory death donors. Nuclei were counterstained with DAPI. Original magnification ×100. DBD, donation after brain death; DCD, donation after circulatory death. ID, immunofluorescence density; NMP, normothermic machine perfusion.

In addition, C3d deposition was examined in human renal biopsies taken after 6 h of NMP (**Figures 9A, B**). C3d deposition was seen in all renal biopsies, independent of the type of donor. However, subgroup analyses demonstrated that renal biopsies derived from DBD donors have significantly more C3d deposition than renal biopsies derived from DCD donors after 6 h of NMP (**Figure 9C**).

### High Levels of Cytokines Are Released During Normothermic Machine Perfusion of Human Discarded Kidneys

**Figure 10:**
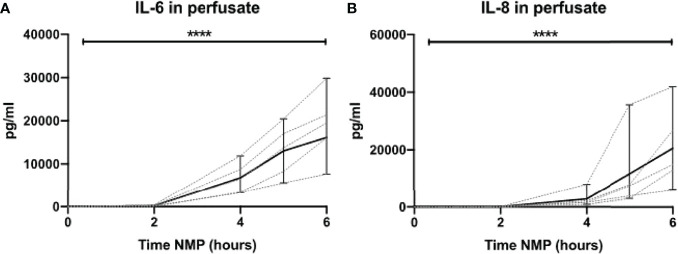
Cytokine perfusate levels during normothermic machine perfusion of human discarded kidneys. Dynamics of pro-inflammatory cytokines **(A)** IL-6 and **(B)** IL-8 in perfusate during normothermic machine perfusion of human discarded kidneys. Cytokine perfusate levels significantly increased during 6 h of normothermic machine perfusion. Dotted lines: increase of cytokine levels in perfusate per individual perfused kidney. Solid lines and bars: median ± interquartile range of cytokine perfusate levels for all kidneys (*n* = 10). ****p < 0.0001. IL-6, interleukin-6; IL-8, interleukin-8; NMP, normothermic machine perfusion.

Lastly, we assessed the release of pro-inflammatory cytokines during NMP of human discarded kidneys. No IL-6 was seen during the first 2 h of NMP. In contrast, there were significantly high levels of IL-6 after 6 h of NMP (**Figure 10A**). Likewise, high levels of IL-8 were observed in perfusate after 6 h of NMP of human discarded kidneys (**Figure 10B**).

## Discussion

In this study, complement activation during NMP was assessed by using porcine and human discarded kidneys. Complement was activated during NMP of porcine kidneys, reflected by increase of complement activation fragments in the perfusate. Furthermore, levels of complement activation during NMP of porcine kidneys positively correlated with pro-inflammatory cytokine levels. Porcine kidneys with high sC5b-9 perfusate levels after 4 h of NMP had a significantly lower creatinine clearance and significantly more histological injury than kidneys with low sC5b-9 perfusate levels. In line with our findings in porcine kidneys, discarded human kidneys showed the same trend of complement activation during NMP. Looking at the different types of donors, kidneys retrieved from brain-dead donors showed significantly higher complement activation during NMP than kidneys retrieved after circulatory death. In addition, kidneys retrieved from brain-dead donors had significantly more renal C3d deposition after NMP. Lastly, an enormous release of pro-inflammatory cytokines IL-6 and IL-8 was seen during NMP of human discarded kidneys.

Complement was immediately activated at the start of NMP. Based on other *ex situ* setups, our results are in line with studies describing complement activation in ECMO, CPB, and HD (19, 20). All these studies reveal an increase in complement C3 activation and sC5b-9 formation within the first 15 min (21, 22). The rapid increase in complement activation products could be due to the initial blood-to-material contact, already described decades ago. This blood-to-material contact could result in immediate adsorption of serum proteins, i.e., complement C3 and immunoglobulin G (19, 23). After the initial activation of complement, levels of complement continued to rise to the end of NMP, consistent with an imbalance between activation and inhibition of the system. The absence of a negative feedback loop to inhibit further complement activation could be the reason for the ongoing complement activation. *In vivo* complement activation is regulated *via* plasma and membrane-bound regulators, which avoid inappropriate complement activation (24). However, complement activation is not regulated by membrane-bound regulators during *ex situ* machine perfusion. In addition, the bio-incompatibility of the NMP system may add to the total activation of complement. Kidney-free NMP confirmed that an initial rise in complement activation is due to blood-to-material contact. However, complement activation levels were significantly higher during NMP compared to the kidney-free system, indicating that the kidney plays an important role in complement activation. Of note, human kidney NMP was performed with a plasma- and leucocyte-free perfusate, but also here complement activation and cytokine production were observed, adding to the notion that the kidney might be regarded as an immunologically active organ on its own (25). Future studies should investigate if complement proteins are produced in the kidney and add fuel to an ongoing complement activation during NMP.

So far, the consequences of complement activation during NMP are unknown. This study shows that complement activation is strongly correlated with the release of the pro-inflammatory cytokines IL-6, IL-8, and TNF. Our results are in line with previous results obtained with porcine kidney NMP, demonstrating the release of pro-inflammatory cytokines IL-6, IL-1β, and IL-18 (26). Interestingly, the dynamics of IL-6 and IL-8 differed from TNF. TNF perfusate levels increase from the start of NMP, while IL-6 and IL-8 perfusate levels started to increase after 2 h of NMP. This might imply that IL-6 and IL-8 might be produced *via* a TNF-dependent pathway (27). Together, these cytokines might propagate further release of adhesion molecules and polymorphonuclear cells contributing to a pro-inflammatory state causing tissue damage (28)..

So far, the consequences of the inflammatory response seen during NMP on renal graft function remains unknown (29). Activation of complement during NMP could theoretically be beneficial, because it could exhaust the complement activation capacity of the renal graft prior to ischemia reperfusion in the recipient. In addition, local complement activation in the renal graft can contribute to the restoration of homeostasis by clearing of cell debris and necrotic sells. However, complement activation during NMP could also have undesirable effects resulting in increased cytokine concentrations and decreased renal function. In our study, we demonstrated that the significant increase in sC5b-9 perfusate levels result in a significant lower creatinine clearance and more histological injury. These results could implicate that complement activation during NMP has adverse effects. However, the human discarded kidneys used in our study are not transplanted into a recipient. Until kidneys after NMP get evaluated in a recipient, no conclusions can be drawn whether activation of complement during NMP is desirable or not. Another potential strategy to investigate the role of complement activation during NMP would be the use of a complement inhibitor, which should be investigated in a future study.

Aiming at translation, we measured complement activation levels during NMP of discarded human kidneys. Like NMP of porcine kidneys, complement activation levels were significantly increased during NMP of human kidneys. In addition, we demonstrated that kidneys retrieved from DBD donors showed a significantly higher C3d/C3 ratio than kidneys retrieved after circulatory death. This might be due to the increased inflammatory response in DBD donors compared to DCD donors. Brain death leads to activation of the immune system, which results in local and systemic inflammation (30). This might lead to an increased inflammatory response in kidneys retrieved from DBD donors during NMP (13, 31). Kidneys retrieved from brain-dead donors might thus specifically benefit from treatment with a complement inhibitor during NMP (18, 32, 33). No differences in complement activation during NMP were seen between preservation with SCS or HMP. This is remarkable, because the outcome of kidneys after HMP is superior to SCS in deceased donor renal transplantation, reflected by the lower incidence of delayed graft function after HMP (34). We speculate that the lack of differences between complement activation in HMP *versus* SCS is due to the attenuated complement activation at low temperatures, which resumes function after rewarming (35). In accordance, multiple studies describe low or no complement activation under other hypothermic conditions (35, 36).

This work has some limitations. First, the number of kidneys included in the human cohort is small and therefore might impact the statistical analyses. In addition, we did not correlate complement activation during NMP of human kidneys to renal injury or renal dysfunction. Creatinine clearance was not possible to assess in human kidneys as the perfusate does not contain creatinine and cannot be added sterile. Therefore, we were not able to include this analysis in this study. Further research should focus on the functional consequences of complement activation during NMP of human kidneys, which is possible only in the recipient after transplantation of an NMP-treated kidney, and test the efficacy of a complement inhibitor during NMP.

In conclusion, this study showed that complement was significantly activated during NMP of both porcine and human kidneys and associated with lower renal function, tissue damage, and inflammation. Complement inhibition during NMP might thus be a promising strategy to reduce renal injury and improve renal graft function prior to transplantation.

## Data Availability Statement

The raw data supporting the conclusions of this article will be made available by the authors, without undue reservation.

## Ethics Statement

Ethical review and approval was not required for the study on human participants in accordance with the local legislation and institutional requirements. Written informed consent for participation was not required for this study in accordance with the national legislation and the institutional requirements. Ethical review and approval was not required for the animal study because Slaughterhouse waste material was used.

## Author Contributions

NJ and LV conducted preclinical porcine experiments, analyzed data, and wrote the manuscript. AA performed human kidney NMP. AM-H and PO assisted with analyses of the samples. TM and SP helped with setting up the complement analyses and supervised the data analysis. NJ, LV, AA, SP, IA, and HL designed the research. SP and HL also had a supervising role. All authors read and approved the manuscript in the submitted version.

## Funding

This study received the following funding: South-Eastern Norway Regional Health Authority (SEP; 2020117).

## Conflict of Interest

The authors declare that the research was conducted in the absence of any commercial or financial relationships that could be construed as a potential conflict of interest.

## Publisher’s Note

All claims expressed in this article are solely those of the authors and do not necessarily represent those of their affiliated organizations, or those of the publisher, the editors and the reviewers. Any product that may be evaluated in this article, or claim that may be made by its manufacturer, is not guaranteed or endorsed by the publisher.
