# Supplementary material for: Complement Is Activated During Normothermic Machine Perfusion of Porcine and Human Discarded Kidneys
Source: Front Immunol. 2022 Jul 13;13:831371. doi: 10.3389/fimmu.2022.831371 (PMC9327788; doi:10.3389/fimmu.2022.831371)
Supplement: Supplementary file 1 [file DataSheet_1.docx]

Supplementary Material

# Supplementary Data

Not applicable

# Supplementary Figures and Tables

## Supplementary Figures

**Supplementary Figure 1. C3a and sC5b-9 perfusate levels for the different preservation groups.** (**A)** C3a and (**B**) sCb5-9 perfusate levels during 4 hours of NMP of porcine kidneys. C3a and sC5b-9 perfusate levels during NMP are measured in kidneys preserved in different ways: static cold storage, hypothermic machine perfusion (HMP) without oxygenation, HMP with 21% oxygen and HMP with 100% oxygen. N = 6 per group. Data are shown as median ± interquartile range. Abbreviations: HMP, hypothermic machine perfusion; NMP, normothermic machine perfusion; sC5b-9, soluble C5b-9.

**Supplementary Figure 2. Dynamics of complement perfusate levels during 4 hours of normothermic machine perfusion with pig blood without kidneys.** Dynamics of (**A**) C3a and (**B**) sC5b-9 perfusate levels during 4 hours of normothermic machine perfusion of heparinized pig blood. Dotted lines: increase of complement perfusate levels per individual perfused kidney. Solid lines and bars: median ± interquartile range complement perfusate levels for all kidneys (n=2) Abbreviations: NMP, normothermic machine perfusion; sC5b-9, soluble C5b-9.

**Supplementary Figure 3. Cytokine perfusate levels for the different preservation groups.** (**A**) IL-6, (**B**) IL-8 and (**C**) TNF perfusate levels during 4 hours of NMP of porcine kidneys. IL-6, IL-8 and TNF perfusate levels are visualized for the different preservation groups: static cold storage, hypothermic machine perfusion (HMP) without oxygenation, HMP with 21% oxygen and HMP with 100% oxygen. N = 6 per group. Data are shown as median ± interquartile range. Abbreviations: HMP, hypothermic machine perfusion; NMP, normothermic machine perfusion.
